# Association Between Tinnitus and Angina Pectoris in U.S. Adults: Evidence from NHANES 2009–2018

**DOI:** 10.3390/audiolres16020035

**Published:** 2026-02-28

**Authors:** Mitra Britton, Ishan Sunilkumar Bhatt

**Affiliations:** 1 Department of Communication Sciences and Disorders, Montclair State University, Montclair, NJ 07043, USA; 2 Department of Communication Sciences and Disorders, University of Iowa, Iowa City, IA 52242, USA; ishan-bhatt@uiowa.edu

**Keywords:** tinnitus, angina pectoris, cardiovascular disease, NHANES

## Abstract

Background/Objectives: Tinnitus has been increasingly associated with cardiovascular disease, and recent phenome-wide analyses have identified angina pectoris as a condition linked to tinnitus. This study aimed to replicate and quantify the association between tinnitus and angina pectoris in a nationally representative U.S. adult sample using NHANES, while adjusting for key demographic, cardiovascular, and tinnitus-related risk factors. Methods: Using data from four NHANES cycles 2009–2018, a cross-sectional analysis was conducted, which included 9185 participants, and used multivariate logistic regression analyses to investigate the association between tinnitus and angina pectoris. Results: Among 9185 adults, angina was associated with higher odds of tinnitus in all models. In the crude model, OR = 3.30 (95% CI: 2.18–4.91, *p* < 0.001); partially adjusted, OR = 1.92 (95% CI: 1.27–2.89, *p* = 0.002); fully adjusted, OR = 1.65 (95% CI: 1.07–2.55, *p* = 0.026). In the fully adjusted model, hearing loss (OR = 4.11), noise exposure (OR = 1.63), current smoking (OR = 1.29), older age (OR = 1.01 per year), and total cholesterol (OR = 1.003 per mg/dL) were additional significant predictors for tinnitus. Conclusions: In this nationally representative sample of U.S. adults, tinnitus was more frequently reported among individuals with a history of angina pectoris, and this association persisted after adjustment for demographic factors, socioeconomic status, hearing loss, noise exposure, smoking, and cardiometabolic comorbidities. These findings support emerging evidence that cardiovascular conditions may be associated with tinnitus, potentially reflecting shared vascular or systemic mechanisms. Given the cross-sectional design, causal inferences cannot be drawn, and the temporal relationship between angina and tinnitus remains unclear. Future longitudinal studies are needed to clarify underlying mechanisms, assess directionality, and determine whether cardiovascular risk modification may have implications for tinnitus prevention or management.

## 1. Introduction

Tinnitus is a common auditory condition characterized by the perception of sound without external auditory stimuli [1]. Over 740 million people worldwide experience tinnitus, with 120 million suffering from its severe form [2]. About 10% to 25% of adults experience tinnitus [3]. Severe tinnitus is associated with several comorbidities, including anxiety, depression, stress, cognitive dysfunction, and insomnia, contributing to a decline in overall quality of life [4,5].

Recent studies indicate a potential association between tinnitus and cardiovascular conditions, as well as their risk factors, such as coronary artery disease, arrhythmia, transient ischemic attack, and hypertension [6,7,8]. A large-scale analysis of the UK Biobank cohort demonstrated a significant association of tinnitus with heightened rates of incident cardiovascular events (hazard ratio (HR) 1.057, 95% confidence interval (CI) 1.017–1.099), and all-cause mortality (HR 1.053, 95% CI 1.003–1.105) [7]. Notably, a phenome-wide co-occurrence association study (PheCAS) of hearing traits in the UK Biobank identified angina pectoris as a significant risk factor for tinnitus [9]. Angina pectoris, defined by chest pain or discomfort, is the primary symptom of ischemic heart disease and the most common clinical presentation of chronic coronary syndrome [10,11]. Angina pectoris was also significantly associated with several hearing-health outcomes, including tinnitus, age-related hearing loss, and speech-in-noise deficits [9]. Subsequent analyses identified angina pectoris as one of the most consistently associated cardiovascular conditions across all tinnitus subtypes, including bothersome, non-bothersome, frequent, occasional, and remitted tinnitus [12]. Collectively, these findings from the UK Biobank provide converging evidence for a significant relationship between tinnitus and angina pectoris. However, independent validation in other populations is lacking.

This study aimed to replicate and quantify the association between tinnitus and angina pectoris using an independent, nationally representative sample from the National Health and Nutrition Examination Survey (NHANES). The analysis adjusted for demographic, socioeconomic, cardiovascular, and tinnitus risk factors, including hearing loss, noise exposure, hypertension, diabetes, and elevated cholesterol [13,14]. Elucidating the relationship between angina pectoris and tinnitus may enhance understanding of shared vascular or neurophysiologic mechanisms, inform preventive and patient-centered management strategies, influence treatment outcomes, and guide future hypothesis-driven research into causal pathways.

## 2. Materials and Methods

### 2.1. Study Design and Population

**Figure 1:**
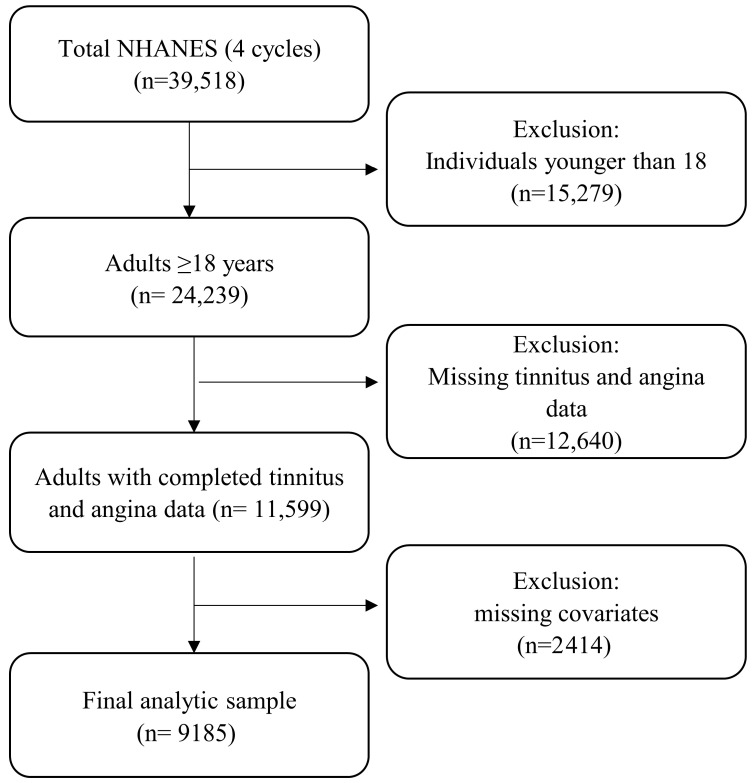
Flowchart of participant selection and exclusion criteria for the analytic sample.

This study utilized NHANES data, a comprehensive survey conducted by the Centers for Disease Control and Prevention (CDC) every 2 years, designed to represent the U.S. civilian, noninstitutionalized population through a stratified, multistage probability sampling approach. For this study, NHANES data from 2009 to 2018 were used, excluding the 2013–2014 cycle because audiology measures relevant to our study were not available. The CDC research ethics review board approves the NHANES procedures and protocols, and all participants provide written informed consent. The screening criteria for the presented study were: (1) aged at least 18 years; (2) information of self-reported tinnitus was available; (3) information of self-reported angina pectoris was available. A total of 39,518 participants from four NHANES cycles were initially considered for this analysis. Participants younger than 18 years (n = 15,279) were excluded, resulting in 24,239 adults. Individuals with missing tinnitus or angina data (n = 12,640) were further excluded, leaving 11,599 participants with complete information on both outcomes. Finally, participants with missing covariate data (n = 2414) were removed, yielding a final analytic sample of 9185 adults (Figure 1).

### 2.2. Assessment of Tinnitus and Angina Pectoris

We utilized NHANES questionnaire data, which assesses an individual’s history of angina pectoris via a self-reported question: “Have you ever been told that you had angina/angina pectoris?” This self-reported assessment has been validated by previous studies, confirming the accuracy of self-reported angina pectoris history [15]. Individuals who answered “yes” were classified as having a history of angina pectoris.

Regarding the tinnitus variable, participants were asked whether, in the past 12 months, they had been bothered by tinnitus, ringing, or buzzing in the ears lasting 5 min or more. Individuals who answered “yes” were considered to have a history of tinnitus. In order to ensure the validity and reliability of our analysis, we excluded any missing responses or those who selected “don’t know” or refused to answer from our sample [16].

### 2.3. Covariates

The covariate list was carefully chosen to statistically control for potential confounders while avoiding the risk of collider bias. Based on the existing literature, we identified several potential confounders, including age, sex, race, education, poverty income ratio (PIR), body mass index (BMI), smoking, hearing loss, noise exposure, hypertension, diabetes, and cholesterol level, for this study [10,13,17]. Race was categorized into five groups, including Mexican American, other Hispanic, non-Hispanic White, non-Hispanic Black, and Other Race-Including Multi-Racial. Education was categorized as high school or below, some college or an associate of arts degree, or a college graduate or above [18]. PIR was included as a continuous variable, with higher values indicating greater family economic status [19]. BMI was calculated by dividing weight in kilograms by height in meters squared (kg/m^2^). Smoking status was determined if a participant answered “yes” to “at least 100 cigarettes smoked in a lifetime” and whether they currently smoke. Smoking status was categorized as never smoker, former smoker, or current smoker [20]. Participants who self-reported their hearing status using the audiometry questionnaire were included in the analysis. Respondents were asked, “Which statement best describes your hearing (without a hearing aid)? Would you say your hearing is excellent, good, that you have a little trouble, moderate trouble, a lot of trouble, or are you deaf?” For this study, self-reported hearing loss was defined as a binary variable. Participants reporting “excellent” or “good” hearing were classified as having no hearing loss, whereas those reporting “a little trouble,” “moderate trouble,” “a lot of trouble,” or being deaf were classified as having hearing loss. The reliability of self-reported hearing loss has been established in previous studies [21]. Noise exposure was defined as a “Yes” response to either job-related or off-work exposure. Job-related exposure was assessed with the question: “Have you ever had a job or combination of jobs where you were exposed to loud sounds or noise for 4 or more hours a day, several days a week?” Off-work exposure was assessed with the question: “Outside of a job, have you ever been exposed to very loud noise or music for 10 or more hours a week?” [22]. Hypertension was defined based on a self-reported question asking participants whether they had ever been told by a doctor or other health professional that they had hypertension, also referred to as high blood pressure. Responding “yes” to this question was considered to have self-reported hypertension. For diabetes, participants who answered “yes” to the question “Other than during pregnancy, have you ever been told by a doctor or health professional that you have diabetes or sugar diabetes?” were classified as having self-reported diabetes. Total cholesterol was measured in mg/dL using laboratory data.

### 2.4. Data Analysis

Statistical analyses were conducted following the NHANES guidelines. NHANES reported an adjusted sample weight for each participant based on the probability of an individual being selected in the sample. This sample weight was applied to all statistical analyses to account for unequal selection and nonresponse probabilities, following the standard NHANES analytical guidelines (https://wwwn.cdc.gov/nchs/nhanes/analyticguidelines.aspx (accessed on 24 January 2026)); thus, our estimates represent the U.S. noninstitutionalized civilian population. We first compared the sample characteristics between participants with and without tinnitus using the chi-squared test for categorical variables and the Wilcoxon rank-sum test for continuous variables. We subsequently used univariate and multivariate logistic regression models applying the appropriate weight to analyze the association between tinnitus and angina pectoris: Model 1 was unadjusted univariable model (Crude model: including only angina), Model 2 was a partially adjusted multivariable model adjusting for age, sex, race, education, PIR, smoking status, and noise exposure, and Model 3 was a multivariable model additionally controlling for hearing loss, BMI, hypertension, diabetes, and total cholesterol. Odds ratios (ORs) and 95% confidence intervals (CI) were calculated with a two-sided *p*-value of 0.05 as statistically significant. All statistical analyses were performed in R (version 4.5.0).

## 3. Results

### 3.1. Participant Characteristics

Table 1 summarizes the demographic details of the participants. In our analysis of 9185 participants, individuals with tinnitus (n = 1516) were older (median age 57 vs. 47 years, *p* < 0.001) and more likely to be male (51.6% vs. 48.4%, *p* = 0.020) compared to those without tinnitus (n = 7669). Racial composition differed significantly between groups, with a higher proportion of non-Hispanic Whites among tinnitus patients (48.7% vs. 37%, *p* < 0.001). Angina pectoris prevalence was significantly higher among those with tinnitus (5.7% vs. 1.9%, *p* < 0.001). Educational status was lower in the tinnitus group, with 50.6% having a high school education or below compared to 41.8% in the no-tinnitus group (*p* < 0.001). Socioeconomic status, measured by the PIR, was lower in tinnitus participants (median 1.81 vs. 2.14, *p* < 0.001). Hearing loss (52.1% vs. 18.1%), noise exposure (53.6% vs. 36.3%), hypertension (50.6% vs. 34%), and diabetes (18.6% vs. 12.7%) were all significantly more prevalent among tinnitus individuals (all *p* < 0.001). Current and former smoking was also higher in the tinnitus group (53% vs. 41%, *p* < 0.001). Median BMI was elevated in tinnitus participants (30 vs. 28, *p* < 0.001). Total cholesterol did not differ significantly between groups (median 190 vs. 189 mg/dL, *p* = 0.051). Table 1 presents the detailed information.

### 3.2. Association Between Tinnitus and Angina Pectoris

In model 1 (crude model), angina was strongly associated with increased odds of tinnitus (OR = 3.30, 95% CI: 2.18–4.91, *p* < 0.001). Table 2 summarizes the weighted logistic regression models evaluating the association between angina and tinnitus for models 2 and 3. In the partially adjusted model (model 2), controlling for age, sex, race, education, PIR, smoking status, and noise exposure, angina remained significantly associated with tinnitus (OR = 1.92, 95% CI: 1.27–2.89, *p* = 0.002). In the fully adjusted model, which additionally accounted for hearing loss, BMI, hypertension, diabetes, and total cholesterol, the association between angina and tinnitus persisted, though attenuated (OR = 1.65, 95% CI: 1.07–2.55, *p* = 0.026).

As shown in Table 2, in the partially adjusted model, older age (OR = 1.03 per year, 95% CI: 1.02–1.03, *p* < 0.001), current smoking (OR = 1.27, 95% CI: 1.05–1.53, *p* = 0.013), and noise exposure (OR = 1.87, 95% CI: 1.51–2.33, *p* < 0.001) were associated with higher odds of tinnitus. Lower odds of tinnitus were observed among Non-Hispanic Black participants (OR = 0.75, 95% CI: 0.59–0.96, *p* = 0.025) and among individuals with a college education or higher (OR = 0.68, 95% CI: 0.50–0.93, *p* = 0.018). In the fully adjusted model, hearing loss was the strongest predictor of tinnitus (OR = 4.11, 95% CI: 3.29–5.15, *p* < 0.001). Noise exposure (OR = 1.63, 95% CI: 1.29–2.06, *p* < 0.001), current smoking (OR = 1.28, 95% CI: 1.06–1.56, *p* = 0.012), and older age (OR = 1.01 per year, 95% CI: 1.00–1.02, *p* < 0.001) also remained significant. Total cholesterol was significantly associated with tinnitus (OR = 1.003, 95% CI: 1.001–1.01, *p* = 0.002). Other demographic and clinical covariates, including sex, race, education, BMI, hypertension, and diabetes, were not statistically significant in the fully adjusted model.

## 4. Discussion

This study aimed to investigate the relationship between tinnitus and angina pectoris in U.S. adults. Our findings showed that having a history of angina pectoris was associated with higher odds of reporting tinnitus. These results are consistent with emerging evidence that tinnitus is associated with cardiovascular disease [23,24,25]. Given the cross-sectional design of this study, causality cannot be inferred. However, genetic analyses using causal inference frameworks suggest that the observed phenotypic association may reflect shared genetic architecture between tinnitus and angina pectoris, rather than a direct causal effect of angina pectoris on tinnitus or tinnitus-related distress [8]. Nevertheless, considering the methodological limitations of genetic epidemiological approaches, future studies should further examine whether this association is driven by shared pathophysiological mechanisms, unmeasured lifestyle- and environment-related factors, overlapping genetic risk, or medication use [26,27,28,29,30].

Arterial stiffness, described as the degradation of elastin and an increase in the collagen levels of arteries, is a key predictor of cardiovascular morbidity and promotes microvascular damage and ischemia [31]. A recent study has reported that carotid artery stiffness was significantly correlated with tinnitus, suggesting that reduced vascular elasticity may impair auditory perfusion [31]. The cochlea receives its blood supply from the labyrinthine artery, a branch of the anterior inferior cerebellar artery that lacks collateral circulation [32]. Consequently, the cochlear microcirculation is particularly vulnerable to disruptions caused by increased arterial stiffness. Consistent with this, tinnitus has been associated with increased risk of ischemic cerebrovascular disease [26] and higher prevalence of vascular comorbidities [6]. These findings support a shared pathophysiological pathway in which microvascular ischemia and arterial stiffening may contribute to tinnitus.

Persistent distress from tinnitus may trigger physiological responses, such as increased sympathetic activity and elevated inflammatory markers, which have been documented in patients with tinnitus and could contribute to an increased risk of cardiovascular disease [6,33]. Heart rate variability analyses suggest that tinnitus is associated with reduced parasympathetic tone and increased sympathetic dominance, with more pronounced autonomic alterations observed in chronic tinnitus compared to acute tinnitus [27]. This autonomic imbalance is associated with elevated heart rate, blood pressure, and vascular resistance, thereby increasing cardiovascular strain. Moreover, altered hemodynamic responses in auditory and nonauditory cortices indicate persistent neural hyperactivity, which may activate stress-related inflammatory pathways and impair endothelial and microvascular function [33,34]. Consistent with this, heightened sympathetic activity and neural inflammation may be shared mechanisms linking tinnitus and ischemic stroke [26]. Additionally, tinnitus may also adversely affect sleep by causing difficulty initiating and maintaining sleep, leading to overall poor sleep quality [35]. Such sleep impairment has been implicated as a contributor to cardiovascular disorders [28]. Pharmacological treatments for cardiovascular diseases may contribute to the observed correlation between cardiovascular diseases and tinnitus [29,36]. A higher prevalence of hypertension treatment was observed among patients with tinnitus compared to controls, suggesting that the ototoxic effects of these medications may contribute to tinnitus development, either independently or in conjunction with hypertension [36]. Additionally, the prevalence of tinnitus was examined in a cohort of 476 patients receiving antihypertensive medication (283 men and 193 women). Overall, 84 patients (17.6%) reported occasional or persistent tinnitus, whereas 392 (82.4%) did not. The incidence was higher among those treated with diuretics, with 72 of 265 patients (27.2%) reporting symptoms. These findings indicate a significant association between diuretic use and tinnitus [29].

An alternative explanation is that tinnitus and angina pectoris are genetically comorbid rather than causally related, as emerging evidence indicates that both conditions are influenced by genetic factors [24,30]. This view is strengthened by the recent observations showing tinnitus severity being significantly phenotypically and genetically correlated with angina pectoris due to shared genetic basis [8]. However, present literature lacks shared genetic analyses to identify candidate tissues and molecular mechanisms underlying their comorbidity. Future research should employ genetic epidemiological methods to elucidate the shared genetic architecture. Finally, the observed association between tinnitus and angina may be influenced by unmeasured lifestyle-related behaviors, including excessive alcohol consumption, unhealthy dietary patterns, and physical inactivity, which may contribute to increased atherosclerotic burden, endothelial dysfunction, and systemic inflammation, thereby elevating the risk of angina. These risk behaviors are more frequently observed among individuals with chronic tinnitus, especially those experiencing sleep disturbances and psychological distress.

We acknowledge that our study had several limitations. First, the cross-sectional design precludes the establishment of a causal relationship or precisely determining the direction of effects between tinnitus and angina pectoris, as there is a possibility of reverse causality. Determining this relationship would require future longitudinal studies. Second, data collection was mainly based on self-reported questionnaires, which may introduce recall bias and influence the reliability of the results. Future studies should consider adding objective measurements to conduct a more accurate assessment.

## 5. Conclusions

In this nationally representative sample of U.S. adults, tinnitus was more frequently reported among individuals with a history of angina pectoris, and this association persisted after adjustment for demographic factors, socioeconomic status, hearing loss, noise exposure, smoking, and cardiometabolic comorbidities. These findings support emerging evidence that cardiovascular conditions may be associated with tinnitus, potentially reflecting shared vascular or systemic mechanisms. Given the cross-sectional design, causal inferences cannot be drawn, and the temporal relationship between angina and tinnitus remains unclear. Future longitudinal studies are needed to clarify underlying mechanisms, assess directionality, and determine whether cardiovascular risk modification may have implications for tinnitus prevention or management.

## Figures and Tables

**Table 1:** Characteristics of the study sample in the NHANES, 2009 to 2018.

Variable	No Tinnitus n = 7669 ^1^	Tinnitus n = 1516 ^1^	*p*-Value ^2^
Angina Pectoris			<0.001
Yes	144 (1.9%)	86 (5.7%)	
No	7525 (98.1%)	1430 (94.3%)	
Age	47 (34–64)	57 (43–69)	<0.001
Sex			0.020
Male	3710 (48.4%)	783 (51.6%)	
Female	3959 (51.6%)	733 (48.4%)	
Race			<0.001
Mexican American	989 (12.9%)	221 (14.6%)	
Other Hispanic	834 (10.9%)	143 (9.4%)	
Non-Hispanic White	2837 (37%)	739 (48.7%)	
Non-Hispanic Black	1753 (22.9%)	278 (18.3%)	
Other Race-Including Multi-Racial	1256 (16.4%)	135 (8.9%)	
Education			<0.001
High school or below	3207 (41.8%)	767 (50.6%)	
Some college or AA degree	2353 (30.7%)	480 (31.7%)	
College graduate or above	2109 (27.5%)	269 (17.7%)	
PIR	2.14 (1.09–4.06)	1.81 (1.01, 3.65)	<0.001
Hearing Loss			<0.001
Yes	1392 (18.1%)	790 (52.1%)	
No	6277 (81.9%)	726(47.9%)	
Noise Exposure			<0.001
Yes	2783 (36.3%)	813 (53.6%)	
No	4886(63.7%)	703 (46.4%)	
Smoking Status			<0.001
Never smoker	4523 (59%)	713 (47%)	
Former smoker	1758 (22.9%)	452 (29.8%)	
Current smoker	1388 (18.1%)	351 (23.2%)	
BMI	27.8 (24.3–32.8)	29.6 (26–34.4)	<0.001
Hypertension			<0.001
Yes	2605 (34%)	767 (50.6%)	
No	5064 (66%)	749 (49.4%)	
Diabetes			<0.001
Yes	978 (12.7%)	282 (18.6%)	
No	6691 (87.3%)	1234 (81.4%)	
Total Cholesterol	189 (163–216)	190 (163, 220)	0.051

^1^ n (%); Median (Q1–Q3); ^2^ Pearson’s Chi-squared test; Wilcoxon rank sum test.

**Table 2:** Weighted partially and fully adjusted logistic regression analyses of the association between angina pectoris and tinnitus among U.S. adults.

Variable	Model 2 OR (95% CI)	*p*-Value	Model 3 OR (95% CI)	*p*-Value
Angina	1.92 (1.27–2.89)	0.002	1.65 (1.07–2.55)	0.026
Age (per year)	1.03 (1.02–1.03)	<0.001	1.01 (1.01–1.02)	<0.001
Sex				
Male	reference	–	reference	–
Female	1.00 (0.82–1.21)	0.974	1.06 (0.88–1.26)	0.550
Race				
Mexican-American	reference	–	reference	–
Non-Hispanic White	1.14 (0.91–1.44)	0.242	1.10 (0.88–1.37)	0.387
Other Hispanic	0.74 (0.55–1.00)	0.047	0.79 (0.58–1.07)	0.121
Non-Hispanic Black	0.75 (0.59–0.96)	0.025	0.84 (0.66–1.07)	0.150
Other/Multi-Racial	0.87 (0.67–1.14)	0.312	0.94 (0.72–1.21)	0.608
Education				
High school or below	reference	–	reference	–
Some college/AA	0.93 (0.75–1.15)	0.474	0.92 (0.73–1.17)	0.449
College graduate+	0.68 (0.50–0.93)	0.018	0.73 (0.53–1.01)	0.048
PIR	1.02 (0.97–1.07)	0.482	1.04 (0.99–1.09)	0.101
Smoking status				
Never	reference	–	reference	–
Former	0.89 (0.70–1.13)	0.323	0.86 (0.69–1.09)	0.218
Current	1.27 (1.05–1.53)	0.013	1.29 (1.06–1.57)	0.012
Noise Exposure (Yes)	1.87 (1.51–2.33)	<0.001	1.63 (1.29–2.06)	<0.001
Hearing Loss (Yes)	–	–	4.11 (3.29–5.15)	<0.001
BMI	–	–	1.01 (1.00–1.03)	0.088
Hypertension (Yes)	–	–	1.19 (0.95–1.49)	0.125
Diabetes (Yes)	–	–	1.03 (0.79–1.34)	0.841
Total Cholesterol	–	–	1.003 (1.001–1.01)	0.002

Partial model: adjusted for age, sex, race, education, PIR, smoking status, and noise exposure; Full model: additionally adjusted for hearing loss, BMI, hypertension, diabetes, and total cholesterol. Odds ratios (OR) and 95% confidence intervals (CI) are presented.

## Data Availability

Data related to NHANES are openly available in a public repository (National Health & Nutrition Examination Survey, https://www.cdc.gov/nchs/nhanes/index.html (accessed on 22 January 2026)).
